# Robust bifunctional aluminium–salen catalysts for the preparation of cyclic carbonates from carbon dioxide and epoxides

**DOI:** 10.3762/bjoc.11.176

**Published:** 2015-09-11

**Authors:** Yuri A Rulev, Zalina Gugkaeva, Victor I Maleev, Michael North, Yuri N Belokon

**Affiliations:** 1 Nesmeyanov Institute of Organoelement Compounds, Moscow 19991, Russia; 2 Green Chemistry Centre of Excellence, Department of Chemistry, University of York, Heslington, York, YO10 5DD, UK

**Keywords:** aluminium, carbon dioxide, cyclic carbonate, epoxide, salen

## Abstract

Two new one-component aluminium-based catalysts for the reaction between epoxides and carbon dioxide have been prepared. The catalysts are composed of aluminium–salen chloride complexes with trialkylammonium groups directly attached to the aromatic rings of the salen ligand. With terminal epoxides, the catalysts induced the formation of cyclic carbonates under mild reaction conditions (25–35 °C; 1–10 bar carbon dioxide pressure). However, with cyclohexene oxide under the same reaction conditions, the same catalysts induced the formation of polycarbonate. The catalysts could be recovered from the reaction mixture and reused.

## Introduction

**Scheme 1:**
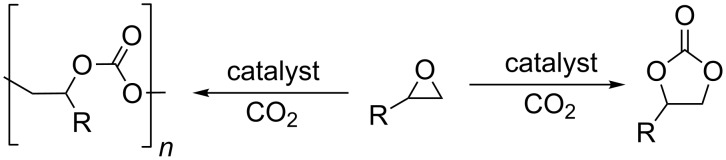
Synthesis of cyclic and polycarbonates.

Carbon dioxide is a renewable and inexpensive carbon source, so great efforts have been directed at developing novel methods for the valorization of this abundant raw material [1]. One way of achieving this goal is to produce cyclic carbonates or polycarbonates from carbon dioxide and the corresponding epoxides (Scheme 1). Cyclic carbonates are an important class of solvents [2] and starting materials in organic synthesis [3–6].

**Figure 1:**
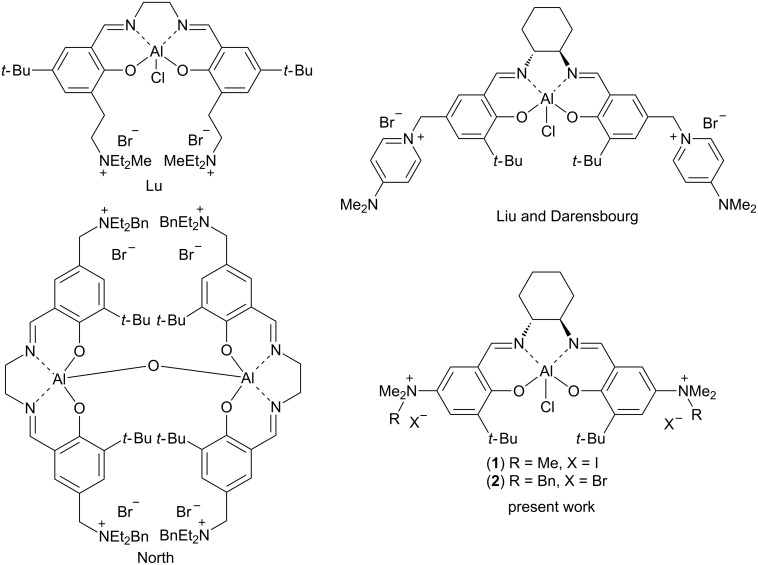
Bifunctional aluminium–salen complexes, including those studied in this work.

Although a significant array of catalysts have been developed for the production of cyclic carbonates [7–9] and polycarbonates [10–11] from carbon dioxide and epoxides, the most developed and privileged set of catalysts are based on Lewis acidic metal–salen complexes. In particular, cobalt(III) and chromium(III) complexes were found to be highly efficient for polycarbonate production [12]. Further modification of the salen moiety by the introduction of basic or ammonium salts through alkyl spacers attached to the salen aromatic rings led to the formation of a family of bifunctional catalysts possessing both Lewis acid and nucleophilic catalysis capability (via the anion in the case of catalysts containing ammonium salts), with a concomitant increase in their activity [12–13]. Recently, more environmentally benign aluminium-based complexes, including salen complexes, have been introduced to catalyse cyclic carbonate production [14]. The performance of these catalysts was also greatly improved by the introduction of bifunctional versions of the catalyst system, combining an electrophilic aluminium centre with an ammonium cation/nucleophilic-counteranion combination within the framework of a single catalytic species as reported by North [15], Liu and Darensbourg [14], and Lu [16] (Figure 1).

Unfortunately, the bifunctional derivatives with an alkyl spacer are not very stable at higher temperatures because of the well-known ammonium salt decomposition pathways including: Zaitsev and Hoffman type eliminations [17–19] and retro-Menschutkin reactions [20–23]. We reasoned that the direct introduction of ammonium moieties onto the aromatic rings of the salen ligands (as in structures **1** and **2**) would greatly stabilize the whole structure by reducing the number of sp^3^-hybridized carbon atoms attached to the nitrogen atoms of the ammonium salts and increasing the steric hindrance around the ammonium salts. Herein, we report the synthesis of two aluminium–salen complexes incorporating quaternary ammonium salts directly attached to the salen ligand and their catalytic activities for the coupling of epoxides and carbon dioxide under solvent free conditions. Catalyst recycling experiments are also reported and show the robustness of this system.

## Results and Discussion

**Scheme 2:**
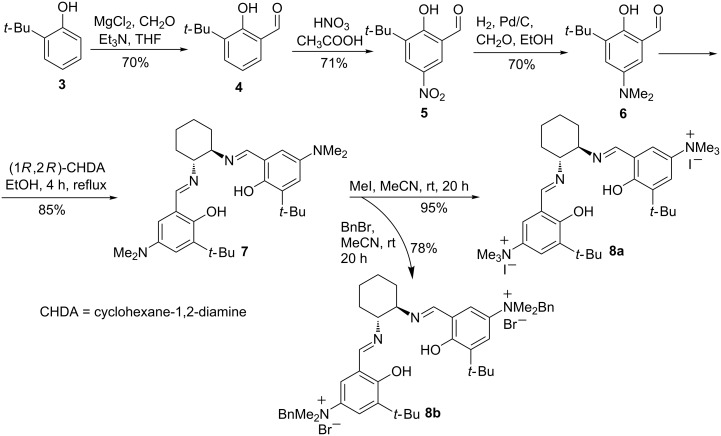
Synthesis of salen ligands **8a** and **8b**.

**Scheme 3:**
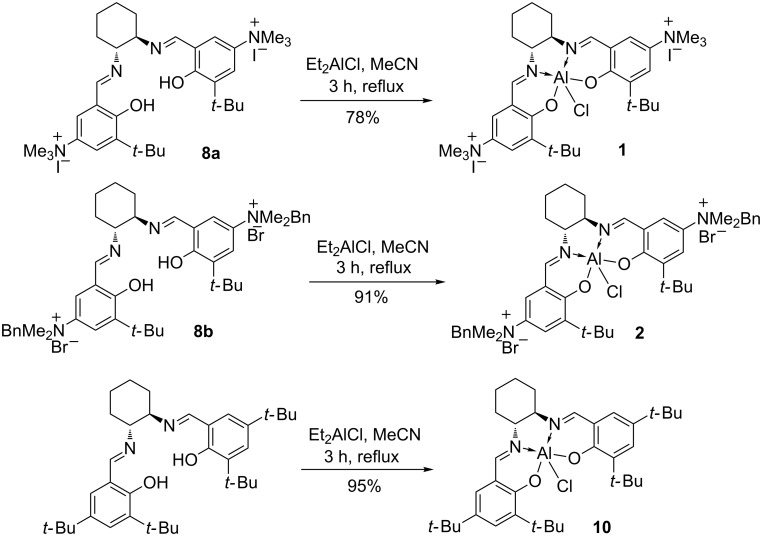
The preparation of aluminum complexes **1**, **2** and **10**.

The preparation of salen ligands **8a** and **8b** was conducted according to Scheme 2, starting from *tert*-butylphenol, which was formylated and then nitrated to produce 5-nitro-3-(*tert*-butyl)salicylaldehyde (**5**) [24–27]. 5-*N,N*-Dimethylamino-3-(*tert*-butyl)salicylaldehyde (**6**) was prepared directly from **5**, using a literature procedure [28–29]. The salen ligand **7** was then obtained in high yield by condensation with (*R,R*)-cyclohexanediamine, according to a known technique [30]. Ligand **7** was efficiently alkylated under mild reaction conditions using either methyl iodide or benzyl bromide, giving the corresponding positively charged salen ligands **8a** and **8b** in 95 and 78% yields respectively. The aluminium–salen complexes were prepared by treating **8a** and **8b** with diethylaluminium chloride (Scheme 3), affording complexes **1** and **2** in 96% and 91% yields respectively. These complexes could be used without any additional purification.

Styrene oxide was used as a model substrate to test the catalytic efficiency of complexes **1** and **2** in the coupling reaction with carbon dioxide. Table 1 summarizes the experimental results. It is apparent from the data that both catalysts are effective at promoting the coupling reaction even at room temperature and 1 bar of carbon dioxide pressure (Table 1, entries 1–4 and 10–12). However, complex **2** was a better catalyst, affording 83% conversion of styrene oxide to the corresponding cyclic carbonate after 24 hours, whereas complex **1** gave only 47% conversion under the same conditions (Table 1, entries 4 and 12). Increasing either the temperature of the reaction or the carbon dioxide pressure had positive effects on the catalytic performance (Table 1, entries 8, 9 and 13).

**Table 1:** Coupling of CO_2_ and styrene oxide promoted by complexes **1**, **2** and **10**.^a^

Entry	Catalyst	Catalyst loading (mol %)	Time (h)	Pressure (bar)	Temperature (°C)	Conversion (%)

1	**1**	0.2	24	1	25	8
2	**1**	1	24	1	25	16
3	**1**	2	24	1	25	40
4	**1**	2.5	24	1	25	47
5^b^	**1**	2.5	3	1	25	8
6^b^	**1**	2.5	6	1	25	17
7^b^	**1**	2.5	24	1	25	72
8	**1**	2.5	24	10	25	70
9	**1**	2.5	24	10	35	100
10	**2**	2.5	3	1	25	14
11	**2**	2.5	6	1	25	43
12	**2**	2.5	24	1	25	83
13	**2**	2.5	24	10	25	100
14	**10**	2.5	24	1	25	5
15^c^	**10**	2.5	24	1	25	80

^a^In neat styrene oxide. ^b^One equivalent of H_2_O and Et_3_N were added to the catalyst. ^c^With 5 mol % tetrabutylammonium iodide as cocatalyst.

**Scheme 4:**
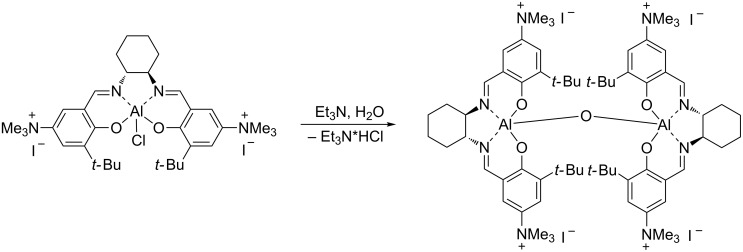
Possible formation of a dinuclear complex from **1** by treatment with H_2_O and Et_3_N.

Furthermore, the performance of catalyst **1** could be improved by adding one equivalent of water and triethylamine relative to the catalyst loading (Table 1, entries 5–7). Presumably, some of complex **1** was converted into a highly active oxygen-bridged aluminium complex in situ, as shown in Scheme 4. High activities for this type of dinuclear complexes have been reported before [31].

In order to prove the bifunctional nature of our catalysts, aluminium complex **10** was prepared (Scheme 3) using 3,5-di-(*tert*-butyl)salicylaldehyde as a starting material. It was found that this catalyst was almost inactive in the reaction of styrene oxide with carbon dioxide (Table 1, entry 14). After addition of tetrabutylammonium iodide (5 mol %) as a cocatalyst, the conversion was increased to 80% (Table 1, entry 15), which was close to the performance of catalyst **2** (Table 1, entry 12). This supports the hypothesis that complexes **1** and **2** are bifunctional catalysts in which both the aluminium centre and the ammonium halide play important catalytic roles.

After finding the optimal reaction conditions for each catalyst, both complexes **1** and **2** were tested with a range of epoxides. These experiments were carried out without added water to allow direct comparison of the two catalysts and to avoid complicating the reaction system. The results are summarized in Table 2. Both catalysts proved to be efficient for coupling both aromatic and aliphatic substrates. In all cases reported in Table 2, cyclic carbonate, catalyst and unreacted epoxide (for entries 10 and 11) were the only species detected by ^1^H NMR spectroscopy of the crude reaction product prior to purification by column chromatography. The moderate yield for propylene oxide (Table 2, entry 6) can be explained by volatility of the starting material under the reaction conditions.

**Table 2:** Coupling of CO_2_ and various epoxides promoted by complexes **1** and **2**.^a^

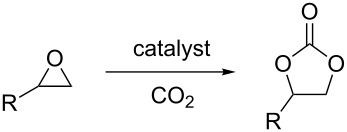				

Entry	Catalyst	R	Conversion^b^ (%)	Yield^c^ (%)

1	**1**	Ph	100	62
2	**1**	CH_2_OPh	100	78
3	**1**	*p*-ClPh	100	95
4	**1**	Bu	100	80
5	**1**	Et	100	78
6	**1**	Me	100	52
7	**1**	CH_2_Cl	100	82
8	**1**	CH_2_OH	100	85
9	**2**	Ph	100	80
10	**2**	CH_2_OPh	64	56
11	**2**	*p*-ClPh	99	84
12	**2**	Bu	100	60
13	**2**	Et	100	71
14	**2**	Me	100	88
15	**2**	CH_2_Cl	100	80
16	**2**	CH_2_OH	100	76

^a^Reaction conditions for catalyst **1**: solvent free, 10 bar pressure of CO_2_, 35 °C, 24 h; for catalyst **2**: solvent free, 10 bar pressure of CO_2_, 25 °C, 24 h. ^b^Determined by ^1^H NMR spectroscopy of the unpurified product. ^c^After purification by column chromatography.

No cyclic carbonate was detected when cyclohexene oxide was used as substrate (Table 3, entries 1–5) and almost no conversion at all was detected in the reaction promoted by complex **1** (Table 3, entries 1 and 2). Catalyst **2** was more active and catalysed the synthesis of the corresponding polycarbonate with 64% conversion at 10 bar (Table 3, entry 4) and 92% at 35 bar carbon dioxide pressure (Table 3, entry 5). Previous reports have indicated that in the presence of a cocatalyst, aluminium–salen complexes can catalyse the formation of either cyclic [32] or polycarbonate [33–34] from cyclohexene oxide, depending on the exact structure of the catalyst and cocatalyst. However, this is the first report of a one-component aluminium–salen-based catalyst for polycyclohexene carbonate synthesis.

**Table 3:** Addition CO_2_ to cyclohexene oxide.^a^

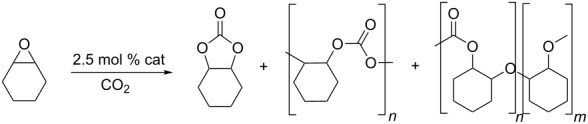						

Entry	Catalyst	Pressure (bar)	Time (h)	Conversion (%)	Polycarbonate (%)	Polyether linkages (%)

1	**1**	10	24	6	6	–
2	**1**	10	111	11	11	–
3	**2**	10	24	8	8	–
4^b^	**2**	10	96	64	57	5
5^b^	**2**	35	96	92	85	7

^a^Neat cyclohexene oxide, temperature: for catalyst **1**, 35 °C; for catalyst **2**, 25 °C, only traces of cyclic carbonates were detected. ^b^The ratio of polycarbonate/polyether was determined from the ^1^H NMR spectrum [11].

**Figure 2:**
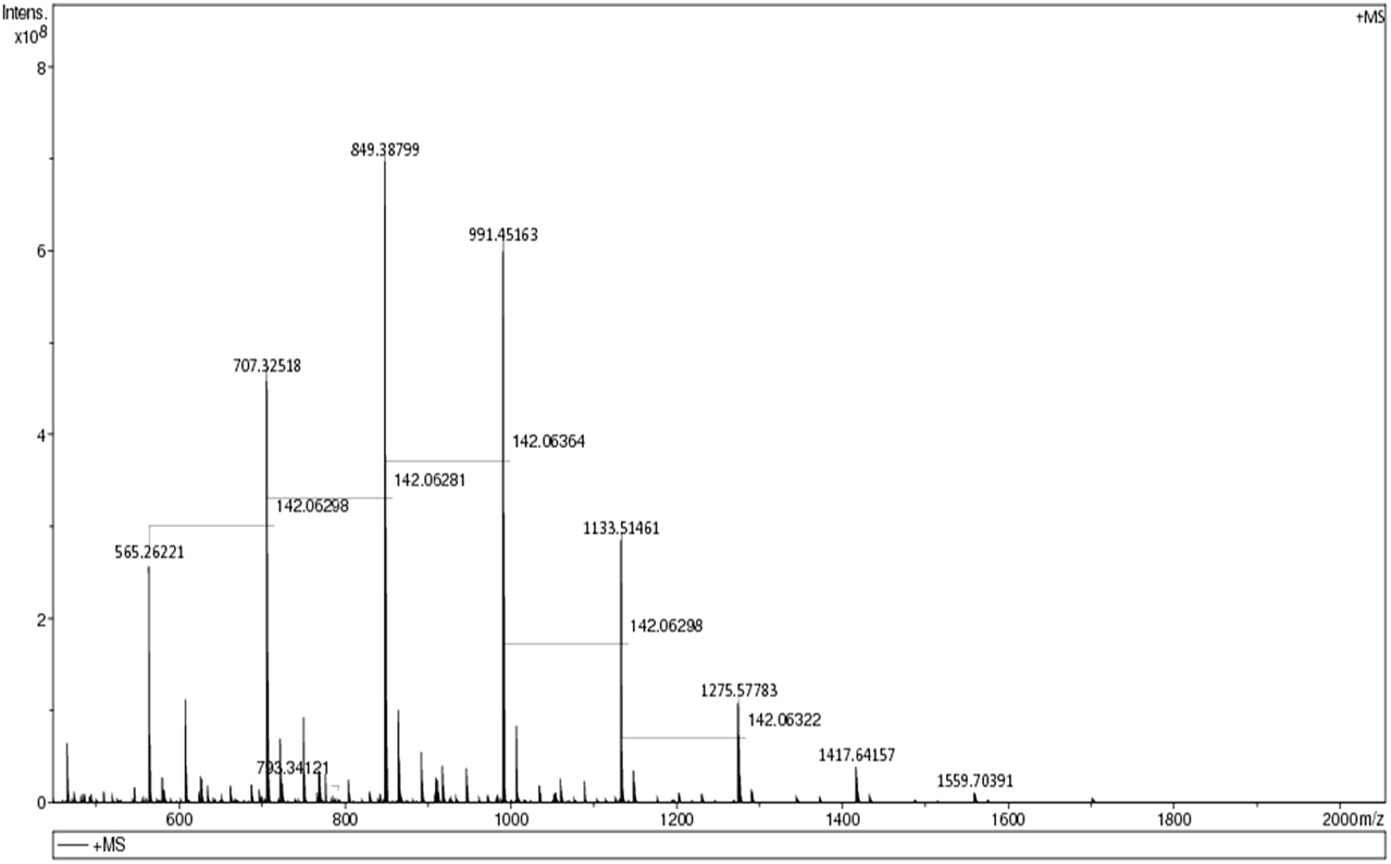
MALDI–TOF spectrum of poly(hexene carbonate) prepared using catalyst **2**. The peak at 565 Daltons corresponds to four ring-opened cyclohexene oxide units, three CO_2_ units, 2 hydrogens (to cap the two terminal oxygens) and a sodium ion. The other peaks are then separated by 142 Daltons corresponding to an additional ring-opened cyclohexene oxide and carbon dioxide.

**Figure 3:**
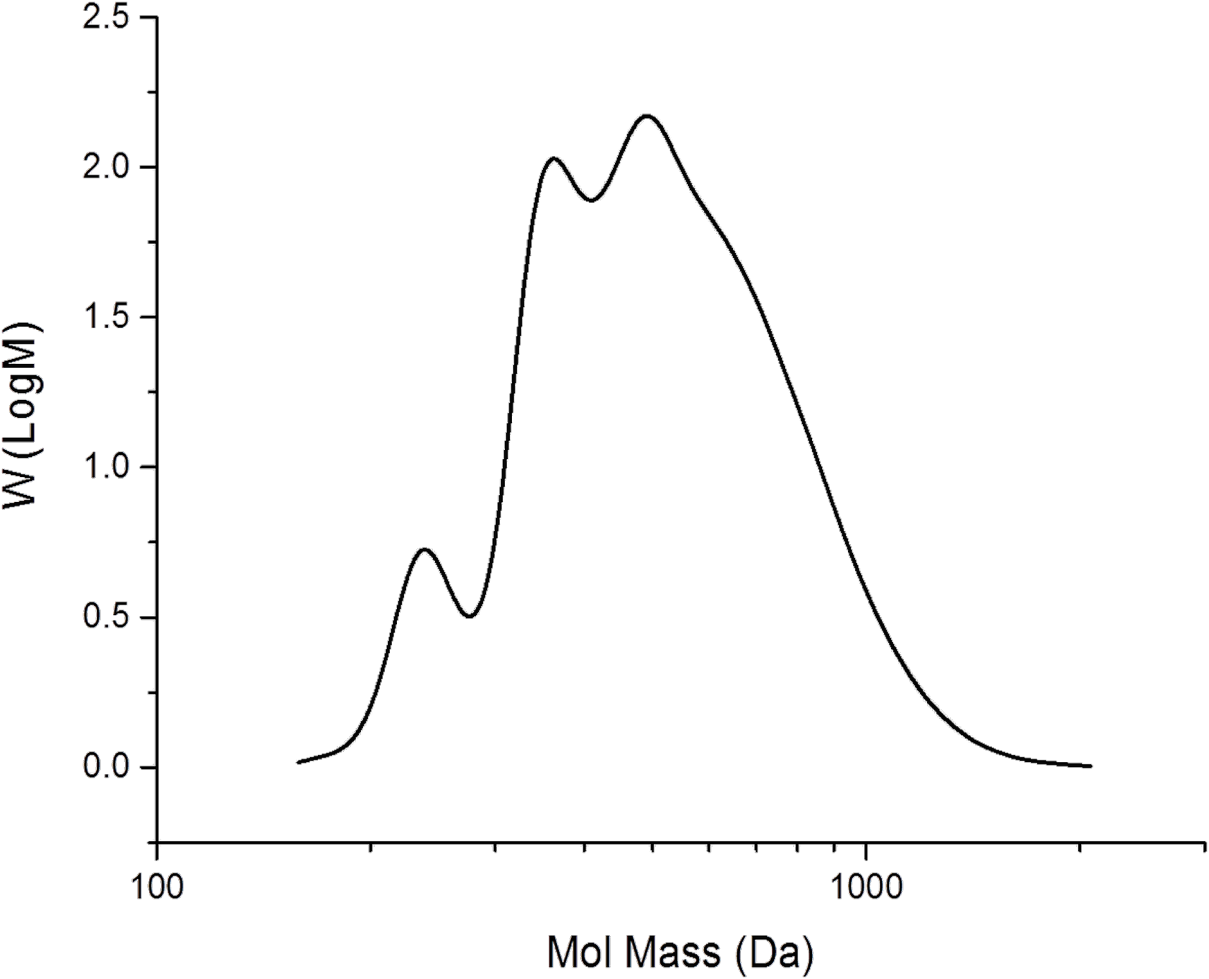
GPC trace of poly(cyclohexene carbonate) prepared using catalyst **2.** The chromatogram was obtained in THF and is referenced to polystyrene standards.

MALDI–TOF mass-spectra data (Figure 2) showed that the polycarbonate consisted of a mixture of oligomers with a range of monomer units (*n* from 4 to 10) with the maximum intensity at *n* = 6. Both ends of the polymer chain are capped with alcohol groups, suggesting that chain-transfer to adventitious moisture occurred during the polymerisation. GPC data (Figure 3) was consistent with the MALDI–TOF data, showing that most of the polymer has a molecular weight between 300 and 1000 Daltons. This type of low molecular weight polycarbonate–polyol is currently attracting much interest associated with its use in sustainable polyurethanes [35].

To show the stability of our catalytic system, catalyst **1** was reused three times. For this purpose the catalyst was precipitated from the reaction mixture by the addition of ether followed by filtration. The catalyst was then dried in vacuo and then reused. The results are summarized in Table 4. As can be seen, there were no significant losses of catalytic activity observed after three catalytic cycles.

**Table 4:** The catalytic activity of recovered catalyst **1**.

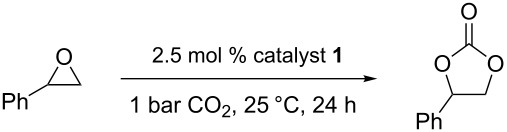	

Cycle	Conversion

1	47
2	45
3	43

## Conclusion

In conclusion, we have developed two new, bifunctional aluminium(salen) catalysts with quaternary ammonium groups directly attached to the aromatic rings. The catalytic system showed high activity in cyclic carbonate formation with a range of substrates. The bifunctional nature of our catalysts was demonstrated by comparing their performance with similar non-modified aluminium complex **10**. In contrast to previously reported aluminium–salen complexes, catalysts **1** and **2** produce polycarbonate rather than cyclic carbonate from cyclohexene oxide.

## Experimental

### Materials

Commercial reagents were used as received unless stated otherwise. Column chromatography was performed using Silica Gel Kieselgel 60 (Merck).

### Instrumentation

^1^H NMR and ^13^C NMR spectra were recorded on Bruker Avance 300 and Bruker Avance III–400 (operating at 300 and 400 MHz for protons, respectively) spectrometers. Optical rotations were measured on a Perkin–Elmer 341 polarimeter in a 5-cm cell. Melting points were determined in open capillary tubes and are uncorrected. Mass spectra were recorded at the University of York Mass Spectrometry Service Unit using ESI and MALDI ionization methods. GPC was carried out using a set (PSS SDV High) of 3 analytical columns (300 × 8 mm, particle diameter 5 µm) of 1000, 10^5^ and 10^6^ Å pore sizes, plus guard column, supplied by Polymer Standards Service GmbH (PSS) installed in a PSS SECcurity GPCsystem. Elution was with tetrahydrofuran at 1 mL/min with a column temperature of 23 °C and detection by refractive index. 20 µL of a 1 mg/mL sample in THF was injected for each measurement and eluted for 40 minutes. Calibration was carried out in the molecular weight range 400–2 × 10^6^ Da using ReadyCal polystyrene standards supplied by Sigma-Aldrich.

### General procedures for compounds (**4**–**10**)

#### 3-(*tert*-Butyl)-2-hydroxybenzaldehyde (**4**)

Prepared as described in previous work [24]. ^1^H NMR (400 MHz, CDCl_3_) δ 11.77 (s, 1H), 9.85 (s, 1H), 7.52 (d, *J* = 7.7 Hz, 1H), 7.39 (d, *J* = 7.7 Hz, 1H), 7.00–6.95 (m, 1H), 1.45 (s, 9H).

#### 3-(*tert*-Butyl)-2-hydroxy-5-nitrobenzaldehyde (**5**)

Prepared by a modified literature procedure [25]. To a stirred solution of 3-(*tert*-butyl)-2-hydroxybenzaldehyde (1.0 g, 4.5 mmol) in glacial acetic acid (20 mL) was added 3.3 M nitric acid (4.0 mL). The solution was heated to reflux for 30 minutes. After cooling to room temperature, the solution was poured onto ice. The resulting precipitate was filtered and washed with water, giving 3-(*tert*-butyl)-2-hydroxy-5-nitrobenzaldehyde (0.9 g, 71%) as a yellow powder. ^1^H NMR (400 MHz, CDCl_3_) δ 12.43 (s, 1H), 9.96 (s, 1H), 8.43–8.35 (m, 2H), 1.45 (s, 9H).

#### 3-(*tert*-Butyl)-5-(dimethylamino)-2-hydroxybenzaldehyde (**6**)

Prepared by a modified literature procedure [28]. Pd/C (10%, 100 mg) was added to a solution of 3-(*tert*-butyl)-2-hydroxy-5-nitrobenzaldehyde **5** (200 mg, 0.9 mmol) and 40% aqueous formaldehyde (4.5 mL) in ethanol (20 mL). The reaction mixture was stirred under 1 bar of hydrogen at room temperature for 24 hours. Then, the Pd/C was removed by filtration on celite, the volume of the filtrate was reduced by half under reduced pressure. Distilled water was added to the solution, resulting in formation of an orange precipitate (140 mg, 70%), which was filtered and washed with water (10 mL) and cold ethanol. ^1^H NMR (400 MHz, CDCl_3_) δ 11.27 (s, 1H), 9.83 (s, 1H), 7.14 (s, 1H), 6.71 (s, 1H), 2.88 (s, 6H), 1.41 (s, 9H); ^13^C NMR (101 MHz, CDCl_3_) δ 197.28, 154.37, 144.05, 138.97, 122.84, 120.34, 114.90, 42.07, 35.14, 29.22; mp 130–131 °C (lit. [28] 134 °C).

#### 6,6'-((1*E*,1'*E*)-((1*R*,2*R*)-Cyclohexane-1,2-diylbis(azanylylidene))bis(methanylylidene))bis(2-(*tert*-butyl)-4-(dimethylamino)phenol) (**7**)

Prepared by a modified literature procedure [30]. A solution of (1*R*,2*R*)-diaminocyclohexane (64 mg, 0.57 mmol) in ethanol (5 mL) was added dropwise to a solution of **6** (250 mg, 1.13 mmol) in ethanol (15 mL). The resulting mixture was refluxed for 4 hours. After cooling to room temperature, distilled water (30 mL) was added. The resulting precipitate was filtered, washed with water and a small amount of cold ethanol to give a yellow powder (250 mg, 85%). ^1^H NMR (400 MHz, CDCl_3_) δ 8.23 (s, 2H), 6.88 (d, *J* = 3.0 Hz, 2H), 6.41 (d, *J* = 3.0 Hz, 2H), 3.35–3.18 (m, 2H), 2.74 (s, 12H), 2.00–1.90 (m, 4H), 1.79–1.72 (m, 2H), 1.47–1.43 (m, 2H), 1.38 (s, 18H); mp 106–110 °C; [α_D_] −225 (*c* 0.01, MeOH); ESIMS *m*/*z*: [M + H]^+^ calcd for C_32_H_48_N_4_O_2_, 521.38; found, 521.39.

#### 5,5'-((1*E*,1'*E*)-((1*R*,2*R*)-Cyclohexane-1,2-diylbis(azanylylidene))bis(methanylylidene))bis(3-(*tert*-butyl)-4-hydroxy-*N,N,N*-trimethylbenzenaminium iodide) (**8a**)

To a solution of **7** (100 mg, 0.192 mmol) in dry acetonitrile (5 mL) was added methyl iodide (271 mg, 1.92 mmol). The solution was stirred at room temperature for 24 hours. Diethyl ether (10 mL) was then added to the solution resulting in formation of a precipitate which was filtered and washed with ether to leave a bright yellow powder (146 mg, 95%). ^1^H NMR (400 MHz, DMSO-*d*_6_) δ 8.59 (s, 1H), 7.85 (d, *J* = 3.2 Hz, 1H), 7.54 (d, *J* = 3.2 Hz, 1H), 3.58 (s, 1H), 3,49 (s, 9H), 1.88 (d, *J* = 11.3 Hz, 1H), 1.78 (s, 1H), 1.64 (s, 1H), 1.46 (s, 1H), 1.32 (s, 9H); ^13^C NMR (75 MHz, CD_3_OD) δ 164.76, 161.55, 139.89, 137.05, 121.25, 120.27, 118.11, 71.57, 56.59, 35.20, 32.47, 28.07, 23.83; mp 206–208 °C; [α_D_] −175 (*c* 0.01, MeOH); ESIMS *m*/*z*: [M]^2+^ calcd for C_34_H_54_N_4_O_2_^2+^, 275.21; found, 275.21.

#### 5,5'-((1*E*,1'*E*)-((1*R*,2*R*)-Cyclohexane-1,2-diylbis(azanylylidene))bis(methanylylidene))bis(*N*-benzyl-3-(*tert*-butyl)-4-hydroxy-*N,N*-dimethyl-benzenaminium bromide) (**8b**)

To a solution of **7** (120 mg, 0.231 mmol) in dry acetonitrile (5 mL) was added benzyl bromide (79 mg, 0.46 mmol). The solution was stirred at room temperature for 24 hours. Diethyl ether (10 mL) was then added to the solution resulting in formation of a precipitate which was filtered and washed with ether to leave a bright yellow powder (165 mg, 78%). ^1^H NMR (400 MHz, CDCl_3_) δ 9.00 (s, 1H), 8.80 (d, *J* = 3.1 Hz, 1H), 7.33–7.27 (m, 1H), 7.19–6.98 (m, 4H), 6.76 (d, *J* = 3.2 Hz, 1H), 5.51–5.37 (s, 2H), 3.98–3.90 (m, 1H), 3.85 (d, *J* = 8.0 Hz, 6H), 2.2–2.1 (m, 1H), 2.0–1.9 (m, 1H), 1.6–1.4 (m, 2H), 1.25 (s, 9H); ^13^C NMR (101 MHz, DMSO-*d*_6_) δ 165.63, 162.37, 139.22, 134.33, 133.01, 130.73, 129.06, 128.87, 123.76, 122.83, 117.57, 72.26, 69.97, 53.21, 35.55, 32.51, 29.37, 29.04, 24.05; mp 140–144 °C; [α_D_] 107 (*c* 0.05, MeOH); ESIMS *m*/*z*: [M]^2+^ calcd for C_46_H_62_N_4_O_2_^2+^, 351.24; found, 351.24.

#### Aluminium–salen complex (**1**)

To a solution of **8a** (113 mg, 0.14 mmol) in dry acetonitrile (5 mL) under argon was added diethylaluminum chloride (0.14 mL, 1 M solution in hexane). The reaction mixture was heated at reflux for 3 hours. The solvent was evaporated under reduced pressure to give a dark yellow powder (95 mg, 78%) which was used without any additional purification. ^1^H NMR (400 MHz, DMSO-*d*_6_) δ 8.46 (s, 1H), 8.10 (s, 1H), 7.66 (s, 1H), 3.55 (s, 9H), 3.38 (s, 1H), 2.55 (s, 1H), 1.94 (s, 1H), 1.50 (s, 9H), 1.44 (s, 1H), 1.33 (s, 1H); mp >300 °C; [α_D_] −109.5 (*c* 0.05, MeOH); ESIMS *m*/*z*: [M]^2+^ calcd for C_34_H_52_AlClN_4_O_2_^2+^, 305.18; found, 296.19 (substitution of Cl by OH); 303.20 (substitution of Cl by OMe).

#### Aluminium–salen complex (**2**)

To a solution of **8b** (140 mg, 0.163 mmol) in dry acetonitrile (5 mL) under argon was added diethylaluminum chloride (0.17 mL, 1 M solution in hexane). The reaction mixture was refluxed for 3 hours. Then, the solvent was evaporated under reduced pressure to give a dark yellow powder (136 mg, 91%) which was used without any additional purification. ^1^H NMR (400 MHz, DMSO-*d*_6_) δ 8.33 (s, 1H), 7.83 (d, *J* = 3.0 Hz, 1H), 7.47 (t, *J* = 8.6 Hz, 1H), 7.40 (s, 1H), 7.28 (t, *J* = 7.7 Hz, 2H), 7.02 (d, *J* = 7.3 Hz, 2H), 4.99 (d, *J* = 7.5 Hz, 2H), 3.55 (d, *J* = 7.3 Hz, 6H), 3.35 (s, 1H) 1.89 (s, 2H), 1.48 (d, *J* = 4.5 Hz, 11H); mp >300 °C; [α_D_] −83.4 (*c* 0.01, MeOH); ESIMS *m*/*z*: [M]^2+^ calcd for C_46_H_60_AlClN_4_O_2_^2+^, 381.21; found, 372.22 (substitution of Cl by OH); 379.20 (substitution of Cl by OMe).

#### Aluminium–salen complex (**10**)

Prepared as described in previous work [29]. ^1^H NMR (400 MHz, DMSO-*d*_6_) δ 8.35 (s, 1H), 7.41 (d, *J* = 2.4 Hz, 1H), 7.36 (d, *J* = 2.4 Hz, 1H), 2.60 (d, *J* = 10.7 Hz, 1H), 1.99–1.90 (s, 1H), 1.53 (s, 9H), 1.40–1.20 (m, 3H), 1.29 (s, 9H).

#### Synthesis of cyclic carbonates

All cyclic carbonate formations were carried out in autoclaves or, in case of 1 bar CO_2_ reactions, in sample vials with a balloon of CO_2_ attached to them. In both cases the reactions were magnetically stirred. After completion of the experiment, the reaction mixture was analysed by ^1^H NMR spectroscopy and passed through a pad of silica to separate the catalyst. In the case of a 100% conversion, CH_2_Cl_2_ was used as the eluent, if the conversion was incomplete then column chromatography was used to purify the compounds (SiO_2_, EtOAc/hexane, 1:3).

**Styrene carbonate:** ^1^H NMR (400 MHz, CDCl_3_) δ 7.44–7.32 (m, 5H), 5.66 (t, *J* = 8.0 Hz, 1H), 4.82–4.73 (m, 1H), 4.37–4.26 (m, 1H); ^13^C NMR (101 MHz, CDCl_3_) δ 155.00, 135.88, 129.80, 129.31, 126.00, 78.11, 71.28.

**4-Chlorostyrene carbonate:** ^1^H NMR (400 MHz, CDCl_3_) δ 7.48–7.25 (m, 4H), 5.65 (t, *J* = 8.0 Hz, 1H), 4.79 (t, *J =* 8.2 Hz, 1H), 4.29 (dd, *J* = 8.6, 7.9 Hz, 1H); ^13^C NMR (101 MHz, CDCl_3_) δ 154.65, 135.85, 134.35, 129.59, 127.37, 77.34, 71.10.

**3-Chloropropylene carbonate:** ^1^H NMR (400 MHz, CDCl_3_) δ 5.02–4.93 (m, 1H), 4.60–4.53 (m, 1H), 4.37 (dd, *J* = 8.9, 5.7 Hz, 1H), 3.82–3.67 (m, 2H); ^13^C NMR (101 MHz, CDCl_3_) δ 154.49, 74.48, 67.06, 44.03.

**3-Phenoxypropylene carbonate:** ^1^H NMR (400 MHz, CDCl_3_) δ 7.37–6.84 (m, 5H), 5.08–4.94 (m, 1H), 4.63–4.46 (m, 2H), 4.30–4.06 (m, 2H); ^13^C NMR (101 MHz, CDCl_3_) δ 157.83, 154.76, 129.78, 122.08, 114.69, 74.20, 66.95, 66.32.

**Propylene carbonate:** ^1^H NMR (400 MHz, CDCl_3_) δ 4.92–4.67 (m, 1H), 4.64–4.38 (m, 1H), 4.07–3.89 (m, 1H), 1.47–1.36 (m, 3H); ^13^C NMR (101 MHz, CDCl_3_) δ 155.22, 73.74, 70.78, 19.45.

**1,2-Hexylene carbonate:** ^1^H NMR (400 MHz, CDCl_3_) δ 4.74–4.59 (m, 1H), 4.53–4.43 (m, 1H), 4.02 (dt, *J* = 9.9, 4.9 Hz, 1H), 1.82–1.57 (m, 2H), 1.46–1.20 (m, 4H), 0.92–0.81 (m, 3H); ^13^C NMR (101 MHz, CDCl_3_) δ 155.24, 77.20, 69.51, 33.59, 26.49, 22.31, 13.86.

**3-Hydroxypropylene carbonate:** ^1^H NMR (400 MHz, CDCl_3_) δ 4.90–4.73 (m, 1H), 4.59–4.39 (m, 2H), 3.96 (dt, *J* = 20.4, 10.2 Hz, 1H), 3.68 (td, *J* =13.1, 5.5 Hz, 1H), 2.84–2.56 (m, 1H); ^13^C NMR (101 MHz, CDCl_3_) δ 155.38, 76.64, 65.85, 61.73.

**1,2-Butylene carbonate:** ^1^H NMR (400 MHz, CDCl_3_) δ 4.71–4.55 (m, 1H), 4.48 (t, *J* = 8.1 Hz, 1H), 4.04 (dd, *J* = 8.4, 7.0 Hz, 1H), 1.85–1.63 (m, 2H), 1.03–0.89 (m, 3H); ^13^C NMR (101 MHz, CDCl_3_) δ 155.27, 78.16, 69.13, 26.95, 8.52.

#### Synthesis of polycyclohexene carbonate

Prepared as reported above for the synthesis of cyclic carbonates at 10–35 bar CO_2_, but without any additional purification of the reaction product. ^1^H NMR (400 MHz, CDCl_3_) δ 4.71–4.56 (broad, 2H), 2.21–2.04 (broad, 4H), 1.79–1.62 (broad, 4H).
